# Flow Behavior Analysis of the Cold Rolling Deformation of an M50 Bearing Ring Based on the Multiscale Finite Element Model

**DOI:** 10.3390/ma18010077

**Published:** 2024-12-27

**Authors:** Wenting Wei, Zheng Liu, Qinglong Liu, Guanghua Zhou, Guocheng Liu, Yanxiong Liu, Lin Hua

**Affiliations:** 1 Hubei Key Laboratory of Advanced Technology for Automotive Components, Wuhan University of Technology, Wuhan 430070, China; wei_wt@whut.edu.cn (W.W.); liu_zh@whut.edu.cn (Z.L.); zhoughjixi@163.com (G.Z.); hualin@whut.edu.cn (L.H.); 2 Hubei Collaborative Innovation Center for Automotive Components Technology, Wuhan University of Technology, Wuhan 430070, China; 3 Hubei Longzhong Laboratory, Xiangyang 441000, China; 4 The State Key Laboratory of Refractories and Metallurgy, Wuhan University of Science and Technology, Wuhan 430081, China; 5 China Railway Construction Heavy Industry Corporation Liminted, Changsha 410000, China; liuql_whut@126.com

**Keywords:** M50 bearing ring, cold rolling, multiscale model, flow behavior

## Abstract

Through the ferrite single-phase parameters of M50 bearing steel obtained based on nanoindentation experiments and the representative volume element (RVE) model established based on the real microstructure of M50, this paper established a multiscale finite element model for the cold ring rolling of M50 and verified its accuracy. The macroscale and mesoscale flow behaviors of the ring during the cold rolling deformation process were examined and explained. The macroscopic flow behavior demonstrated that the stress distribution was uniform following rolling. The equivalent plastic strain (PEEQ) grew stepwise over time, with the raceway showing the highest PEEQ. The mesoscopic simulation revealed that the stress was concentrated in the cementite, and the maximum occurred at the junction of the ferrite and cementite. The largest PEEQ was found in the ferrite matrix positioned between the two adjacent cementites. The cementite flew with the deformation of the ferrite. The radial displacement of the cementite decreased from the edge of the raceway to both ends and decreased from the inner to the outer surface. Its axial displacement was basically the same on the inner surface and decreased from the inner to the outer surface. Its circumferential displacement decreased from the inner and outer surfaces to the intermediate thickness region.

## 1. Introduction

M50 bearing steel is a widely utilized high-temperature bearing steel in the aerospace industry [1,2]. M50 is classified as a high-carbon bearing steel, and the presence of chromium (Cr), molybdenum (Mo), and vanadium (V) within its composition facilitates the formation of various types of cementite, which are diffusely distributed throughout the matrix [3]. This characteristic is crucial for ensuring its exceptional red hardness and fatigue life. Ring rolling technology is an advanced and efficient continuous partial plastic forming technology, which is mainly used for manufacturing seamless rings. Compared with the traditional hot-rolled rings, the products obtained by cold rolling have good surface quality, high dimensional accuracy, high mechanical strength, and longer fatigue life. With the wide application of the cold ring rolling process on 100Cr6 bearing steel, in recent years, there are also scholars who study using the cold ring rolling process to process M50 bearing steel [4,5,6,7]. Through experimentation and numerical simulation, Baoshou Sun [4] examined the impact of mandrel roll feeding rate during cold ring rolling of GCr15 bearing steel. Additionally, there was good agreement between the two approaches’ outcomes. S. Deng et al. [8] used a combination of cold-ring-rolled finite element simulation and testing to study GCr15 bearing steel. They examined the evolution of the weave after different deformation rates and the microstructural changes in cold ring rolling. Using experimental techniques, Feng Wang et al. [6] examined the microstructure evolution and tempering transformation kinetics of M50 bearing steel during cold ring rolling and came to insightful findings.

In the context of high-carbon bearing steels, it has become increasingly acknowledged that cold deformation can induce damage or even microcracking within the material [9,10,11]. If the microcracks resulting from cold deformation are not entirely mitigated through heat treatment and machining processes, they may lead to significant issues during service, potentially serving as a critical factor in ring failure [12,13,14,15]. Current research on cold ring rolling technology primarily focuses on macroflow behavior, stress–strain distribution, and formability, often neglecting the meso- and microlevels. Therefore, this study of the second phase and the matrix in the mesolevel change in cold rolling technology has great importance for the bearing rolling process and the prevention of bearing cracks.

Multiscale approaches are widely used in the research of metallic and composite materials as an important way to bridge macroscale and mesoscale processes. Piotr Macioł et al. [16] explored the potential for parallelizing multiscale models within the agile multiscale methodology framework by integrating finite element method (FEM)-based continuous macroscopic models with concurrently computed mesoscale models. Yutai Su et al. [17] introduced a novel rapid method for generating representative volume elements (RVEs) of randomly distributed metal matrix nanocomposites characterized by quasi-continuous volume distributions, utilizing a combination of the Gaussian filtering algorithm and cutting quantile functions, and they subsequently validated the accuracy of their model. Lingwei Kong et al. [18] developed a new constitutive model that accounts for the mesoscopic damage and plasticity mechanisms of quasi-brittle materials through the coupling of micro-planes and micromechanics. Zhang et al. [19] proposed a multiscale MCCPFEM framework to simulate microscale thermal interface grooving (TIG) welding and mesoscale deformation anisotropy, effectively capturing and analyzing the interplay between deformation anisotropy and TIG-induced tissue evolution during the isothermal compression of the IMI834 alloy with lamellar clusters. For M50 bearing steel, multiscale modeling can solve the problems of the accurate characterization of its microstructure, controlling the morphology and distribution of cementite during the annealing process as well as reducing the number of physical experiments and saving costs. Consequently, multiscale analysis proves to be particularly valuable for investigating the connections between macroscopic and mesoscale phenomena in the cold rolling process of M50 bearing rings. However, there are still very few reports that specifically cover this subject.

The RVE is defined as a mesoscopic volume element within a macroscopic body of material. Through the study of this element, the linkage between the macroscopic and mesoscopic deformation mechanisms of the material is established. In metallic materials represented by dual-phase steels, the method of establishing RVE models based on the real microstructure of the material has matured, and the accuracy of the method has been verified [20,21]. However, in metal matrix composites and in metallic materials, such as GCr15 and M50, where granular phases are present, conventional RVE modeling is generally based on microscopic characterization statistics and stochastic algorithms [17,22]. Although this method is able to reflect the interaction of multiple meso-structures under macro-deformation, it ignores the microstructural features of the material and does not provide a completely accurate description of the mesoscale changes in the material. Therefore, an RVE model that accurately reflects the meso-structure of the second phase is needed in the multiscale analysis of such materials.

This paper presented a multiscale approach to the investigation of the cold ring rolling process. Firstly, a macro-finite element model of cold ring rolling was developed, grounded in actual working conditions. Subsequently, a sub-model was employed to facilitate the transfer of mechanical responses between the macroscale and mesoscale. Then, utilizing image processing techniques, we established an RVE that accurately reflected the mesoscopic changes. This framework enabled the multiscale numerical simulation of the cold ring rolling process for M50 bearing steel, allowing for an in-depth examination of the macro- and mesoflow behaviors associated with the cold ring rolling process.

## 2. Materials and Methods

### 2.1. M50 Bearing Steel

**Figure 1:**
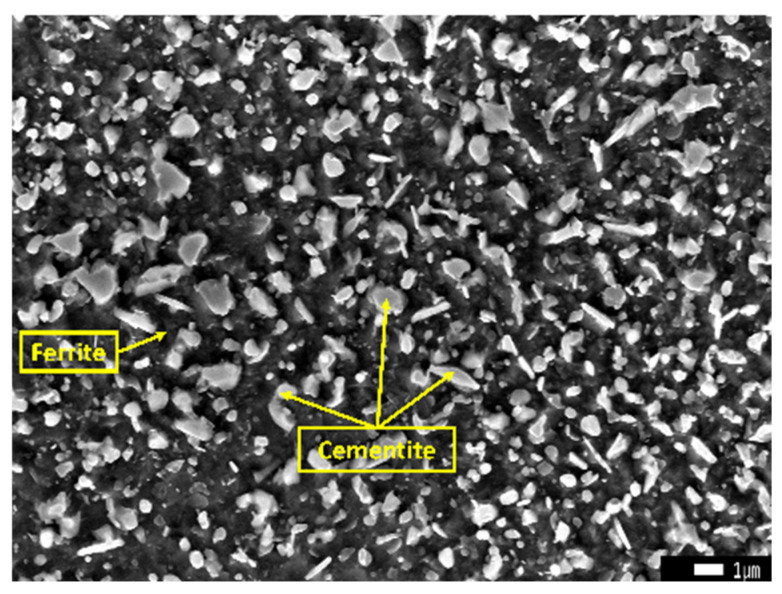
SEM image of M50 bearing steel in spheroidized annealed condition.

This research employed ring blanks made from commercially accessible M50 bearing steel, with the chemical compositions of the initial material detailed in Table 1. The microstructural characteristics of the bearing steel, examined using a scanning electron microscope (SEM) (JSM-IT800(HL), Nippon Electronics Corporation, Tokyo, Japan) after undergoing spheroidal annealing heat treatment, are depicted in Figure 1. In this figure, the bright white regions represented cementite, whereas the other areas were classified as ferrite.

### 2.2. Macro-Finite Element Modelling

**Figure 2:**
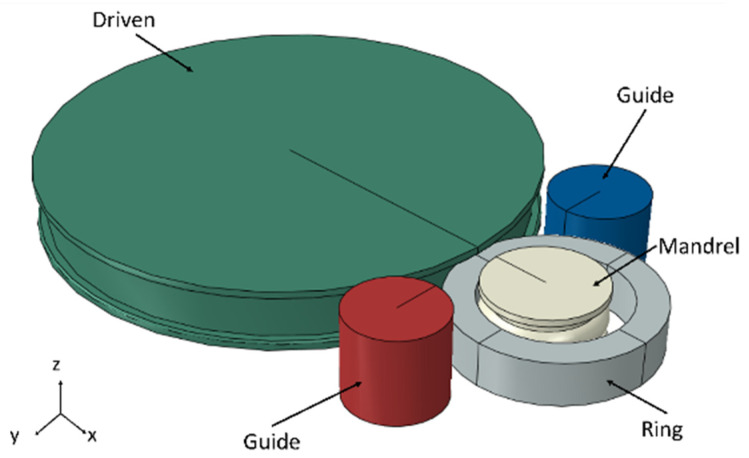
Macrocold rolling finite element model of the M50 bearing ring.

In this study, the commercial finite element software ABAQUS (ABAQUS 614) was employed to develop a macrocold rolling finite element model of the M50 bearing ring, as shown in Figure 2. The model components, including the driven roller, mandrel roller, ring blank, and guide roller, were constructed based on parameters derived from actual working conditions, as detailed in Table 2. However, the stiffness of the mold is often much larger than the stiffness of the ring material, so the mold was generally set as a rigid body in the simulation. The driven roller was constrained to rotate solely around the z-axis, while the mandrel roller was permitted to rotate under applied forces and translate in the negative direction of the x-axis. The guide rollers were capable of translation within the XOY plane and rotation around the z-axis. The friction coefficient between the driven roller, mandrel roller, and ring was set to 0.3, whereas the interface between the guide roller and the ring was assumed to be smooth. For the ring blank, a mesh size of 0.5 mm was utilized, employing an eight-node linear hexahedron (C3D8R) mesh type, which incorporates linear interpolation and selective reduced integration. In addition, the Arbitrary Lagrangian–Eulerian (ALE) adaptive mesh technique is applied to the ring components to automatically adjust the coarseness and refinement of the mesh to achieve more accurate results and higher computational efficiency. As for the mold, no meshing is required for it, as it is set as a rigid body.

### 2.3. Meso-Finite Element Modelling

**Figure 3:**
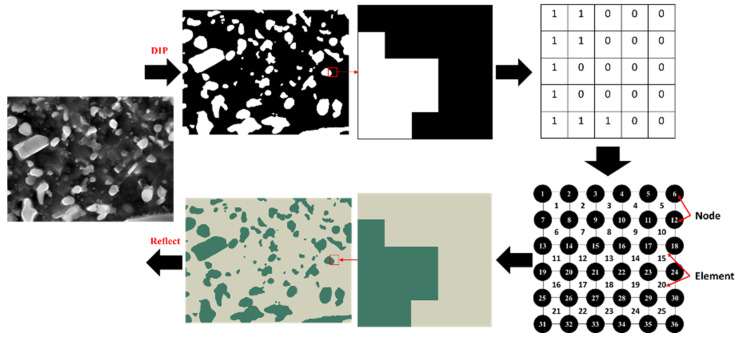
The schematic diagram for constructing the RVE model.

This paper presented an RVE model derived from the actual microstructure of the material, which effectively reflected the meso-structure of cementite and provided a more accurate representation of the correlations involved. Figure 3 shows the schematic diagram for constructing the RVE model. The SEM image of M50 bearing steel was systematically processed using digital image processing (DIP) techniques at the pixel level to differentiate the matrix from the second phase and to compile the pixel point information matrix. Each pixel point was treated as an element with a size of 0.005 µm, thereby establishing a connection between elements and nodes. To distinguish the matrix from the second phase, separate sets were created, and the RVE model that corresponded to the actual microstructure was developed and formatted as an input file. Finally, the generated input file was imported into ABAQUS software to create the corresponding model.

**Figure 4:**
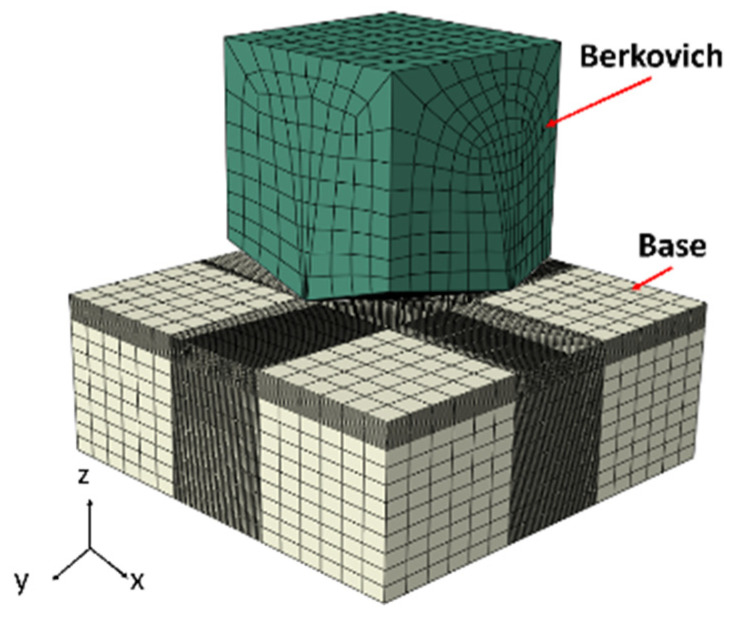
Three-dimensional finite element modeling of nanoindentation.

To ensure the accuracy of the RVE model, it is essential to obtain single-phase stress–strain curves for M50 bearing steel, specifically for the ferrite and cementite phases. The mechanical properties of ferrite can be determined through nanoindentation experiments and inverse analyses of the load–displacement curves [23,24,25,26]. In contrast, cementite is typically regarded as an elastic or brittle material due to its minimal or negligible deformation during the loading process [27,28]. The mechanical parameters of ferrite were derived using the method established by Oliver and Pharr [29]. A commercial indenter (TI980 (NULL, Hangzhou, China)) with a Berkovich-type diamond (1140 GPa modulus, 0.07 Poisson’s ratio) was used in nanoindentation experiments to produce load–displacement (P-H) curves. The indenter had a radius of 50 nm, an inclination angle of 65.3°, and an indentation depth of 150 nm. The stress–strain curves for the ferrite single phase were calculated from the experimental load–depth data. Subsequently, a three-dimensional finite element model of the nanoindentation process was established in accordance with the experimental conditions, as illustrated in Figure 4. In this model, the indenter was represented as a three-dimensional discrete rigid body, constrained to move −150 nm along the z-axis. The base’s lower end, which was assigned the single-phase parameters obtained from the aforementioned calculations, was fixed in place. Mesh refinement was conducted in the contact region, utilizing a refined mesh size of 100 nm and employing the C3D8R element type. Finally, a comparison was made between the P-H curves obtained from both experimental and simulation data to ascertain the stress–strain curve of the ferrite phase. Detailed results are presented in Section 3.2.

**Figure 5:**
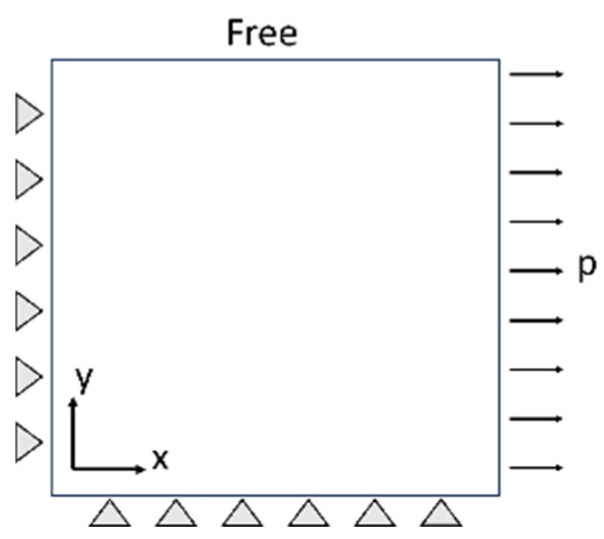
Schematic view of the RVE unidirectional stretching condition.

Following the construction of the RVE model, it is imperative to verify the model’s reliability. Ma, S. M. [30,31], Zhao, K. [32], and colleagues assessed the validity of the RVE model by applying a unidirectional tensile boundary condition and analyzing the discrepancies between the resulting stress–strain curve and the macroscopic tensile curve. As illustrated in Figure 5, symmetry boundary conditions were applied to the left node of the RVE model, while displacement variations were introduced on the right side. The stress–strain curves were computed using a first-order homogenization strategy, after which these curves were compared to the macroscopic tensile curves to ascertain the model’s reliability. Detailed results are presented in Section 3.2.

**Figure 6:**
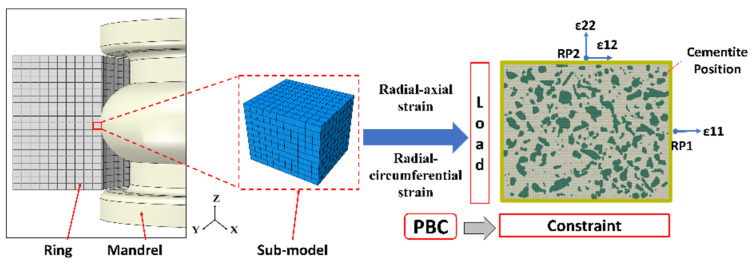
Multiscale finite element model.

After verifying the reliability of the RVE model under tensile conditions, a multiscale numerical simulation of the cold rolling process for the ring can be conducted. The multiscale finite element modeling is shown in Figure 6. The sub-model was utilized to extract and refine the macroscopic element individually and to establish links between macroscopic and mesoscale elements. The mesoscale model utilized the element strains from the sub-model as the driving conditions. Specifically, the three-dimensional strain was decomposed into a radial–axial strain and a radial–circumferential strain; the change in the RVE in the radial–axial direction was mainly observed, and the change in the radial–circumferential direction was used to support the illustration. The strains ε11, ε12, and ε22 in the radial–axial and radial–circumferential directions, transmitted by the sub-model, were applied as loads to the RVE model, individually. The selection of the RVE model was predicated on an analysis of the images captured under SEM in the preceding period, thereby facilitating the acquisition of the composition and distribution characteristics of the material’s secondary phase. Subsequently, a representative image was selected from the existing array of images to serve as the foundation for developing the RVE model. A 4-node bilinear plane strain quadrilateral element was selected for the mesh type to effectively simulate the plane stress state. Constraints were implemented to connect the nodes at the edges of the RVE to a reference point, facilitating the application of loads. The establishment of periodic boundary conditions (PBCs) allowed the RVE model to maintain consistent deformation states for the upper and lower elements, as well as the left and right elements, thereby enabling the simulation of the material’s actual flow behavior. Python software (Python 3.10) was used to carry out batch operations on the input files for all of the previously listed procedures. Furthermore, the locations of the cementite, as indicated in the figure, were selected for subsequent flow analyses.

## 3. Results and Discussion

### 3.1. Macroscopic Flow Behavior Analysis of M50 Cold-Rolled Bearing Rings

**Figure 7:**
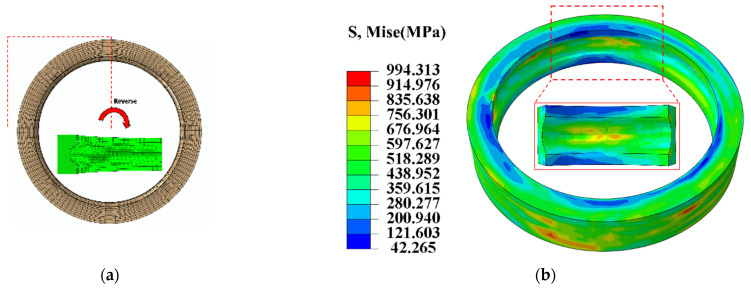
Schematic view of macrocold rolling deformation results of the M50 bearing ring. (**a**) Axial view of the forging at the end of rolling; (**b**) stress distribution of the ring.

Figure 7 presents the schematic view illustrating the macrocold rolling deformation results of the M50 bearing ring. Specifically, Figure 7a depicts the axial view of the forging at the end of the rolling process, and a quarter of the ring was intercepted for display. Figure 7b illustrates the stress distribution at the same time period as well as the partial magnification at the raceway. Table 3 provides a comparison of the simulated and expected dimensions of the forging post-rolling. Analysis of Figure 7a and Table 3 indicates that the roundness of the forging at the end of the rolling process is satisfactory, with minimal discrepancies between the simulation results and the anticipated dimensions. Comparing the expected dimensions to the highest outer direction (OD) error, the difference is only 0.375%. The inner diameter (ID) inaccuracy can be as much as 0.32%. There is just a 0.5% maximum inaccuracy in the ring thickness. Furthermore, as evidenced in Figure 7b, the stress distribution across the ring component at the conclusion of the rolling process was found to be uniform. The minimum stress values were observed at the upper and lower end surfaces of the ring, whereas the maximum stress values were noted sporadically at the outer surface of the ring and the raceway, with negligible evidence of stress concentration.

**Figure 8:**
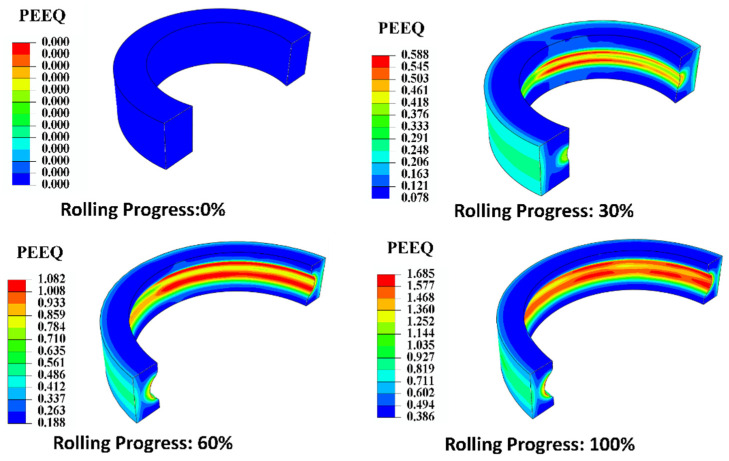
Distribution of the PEEQ during the cold rolling of the ring.

Figure 8 illustrates the equivalent plastic strain (PEEQ) distribution during the cold rolling of the ring. During the initial phase of rolling, the ring was clearly subject to forces from the driven roll as well as the convex mold of the mandrel roll. This interaction led to the emergence of the PEEQ, primarily on the outer surface of the ring and at the raceway. As the mandrel roll advanced, it established full contact with the inner surface of the ring, resulting in variations in the PEEQ on both the outer surface and the entire inner surface of the ring, which subsequently extended into the intermediate thickness region. By the conclusion of the rolling process, the distribution of the PEEQ within the ring was characterized by a non-uniform pattern, exhibiting a gradual transition from lower to higher strain values and moving from the middle thickness region towards the surface region. The maximum PEEQ was observed at the raceway on the inner surface of the ring, while the minimum PEEQ was found in the middle region of the upper-end face of the ring.

**Figure 9:**
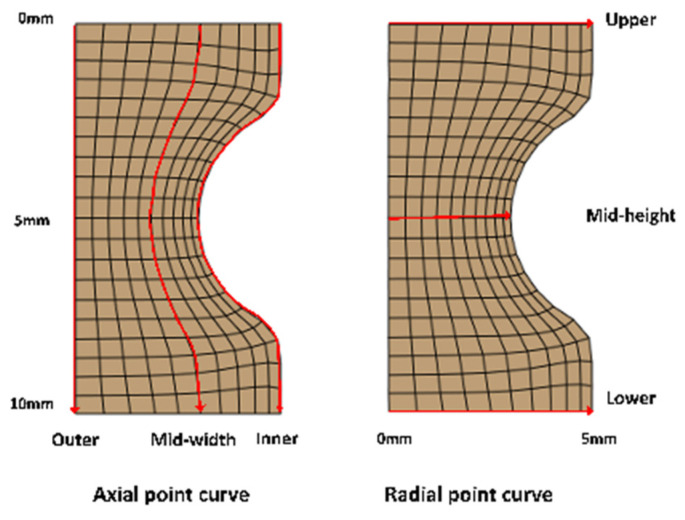
PEEQ cross-section pickup point path diagram at the end of rolling.

**Figure 10:**
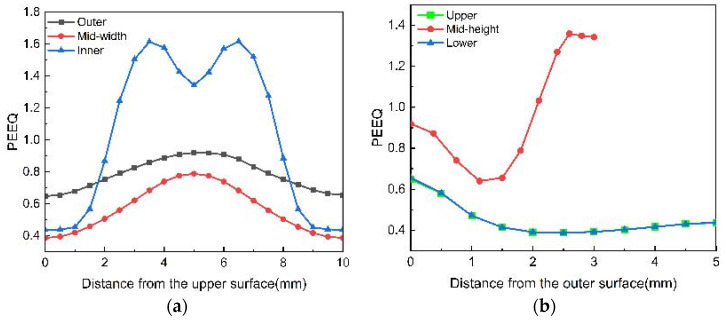
Distribution of the PEEQ after rolling. (**a**) Axial distribution; (**b**) radial distribution.

Figure 9 illustrates the trajectory of pickup points utilized for the analysis of the PEEQ at the end of the rolling process. This analysis encompassed the axial selection of locations at the upper-end face, the mid-height region, and the lower-end face of the ring, as well as the radial selection of positions at the outer surface, the mid-thickness region, and the inner surface of the ring. The variation curves of the PEEQ for the aforementioned locations are presented in Figure 10. It was observed that the PEEQ at each position on the ring followed a descending order. The raceway of the inner surface exhibits the highest strain, followed by the outer surface and then the intermediate thickness region. Additionally, compared to the top and lower-end face sections, the PEEQ in the ring’s mid-height zone was higher. In the radial direction, the disparity in diameter between the driven roll and the mandrel roll resulted in a varying PEEQ on the inner and outer surfaces of the ring. In the axial direction, the presence of a closed hole pattern, along with the symmetry of the mandrel roller at the top and bottom, led to a PEEQ that was symmetrical about the mid-height. Notably, the PEEQ at the raceways on the inner surface was significantly greater than observed at other locations.

**Figure 11:**
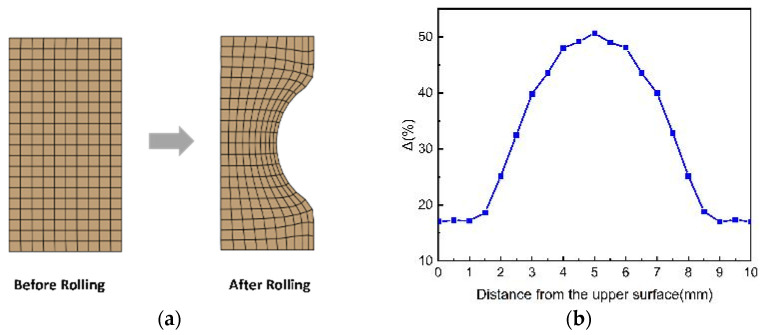
Radial deformation of the ring. (**a**) Cross-section of the ring before and after rolling; (**b**) curve of the radial deformation of the ring.

At the conclusion of the rolling process, the radial deformation of the ring serves as an indicator of the extent of deformation experienced by the ring, which is crucial for directing the rolling operation. Figure 11a illustrates the radial–axial cross-section of the ring component both prior to and following the rolling process. The radial deformation at each position at the end of rolling was determined based on the radial dimensions of the ring, with the calculation formula provided in Equation (1).

(1) $$\Delta =\frac{\left|B-B_{0}\right|}{B_{0}}\times 100\%$$

where Δ is the radial deformation, $B_{0}$ is the ring thickness before rolling, and B is the ring thickness after rolling.

Figure 11b presents the curve depicting the variation in radial deformation of the ring subsequent to the rolling process. It was apparent that the radial deformations of the ring displayed considerable disparities due to the influence of the raceway; nonetheless, these deformations maintained a symmetrical profile along the central height region, a characteristic attributed to the design of the closed hole pattern. The radial deformation exhibited a gradual decline from the raceway towards both lateral extremities of the ring. The peak deformation was noted at the mid-height of the ring, reaching a maximum of 49%, whereas the minimum deformation was observed at the end face of the ring, recorded at 17%.

**Figure 12:**
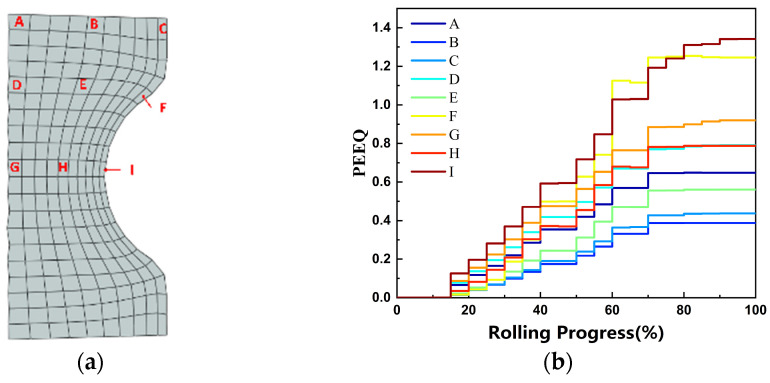
PEEQ curve of the ring. (**a**) Element selection position; (**b**) PEEQ gradient change curves.

To accurately depict the progression of the PEEQ at various feature locations on the ring during the rolling process, nine feature elements were identified, as shown in Figure 12a, corresponding to points A through I. Figure 12b illustrates the changes in the PEEQ for each feature element throughout the rolling process. It was apparent that due to the presence of a reserved gap, no PEEQ was observed at the initiation of the rolling process. As the mandrel roller progressed, the PEEQ in each section of the ring exhibited a stepwise increase, primarily resulting from the continuous movement of the plastic deformation zone within the ring. Upon the ring’s entry into the radial hole pattern, substantial plastic deformation occurred, as evidenced by the ascending steps of deformation depicted in the figure. In contrast, minimal plastic deformation was noted when the ring exited the hole pattern, which corresponded to the horizontal phase of the effect transformation illustrated in the figure. Additionally, the PEEQ gradient curves revealed that at the conclusion of the rolling process, the PEEQ was maximized at the raceway (corresponding to points F and I in the figure) and minimized at the mid-thickness zone of the upper-end face and the non-raceway zone (corresponding to points B and C in the figure).

### 3.2. Mesoscopic Flow Behavior Analysis of M50 Cold-Rolled Bearing Rings

**Figure 13:**
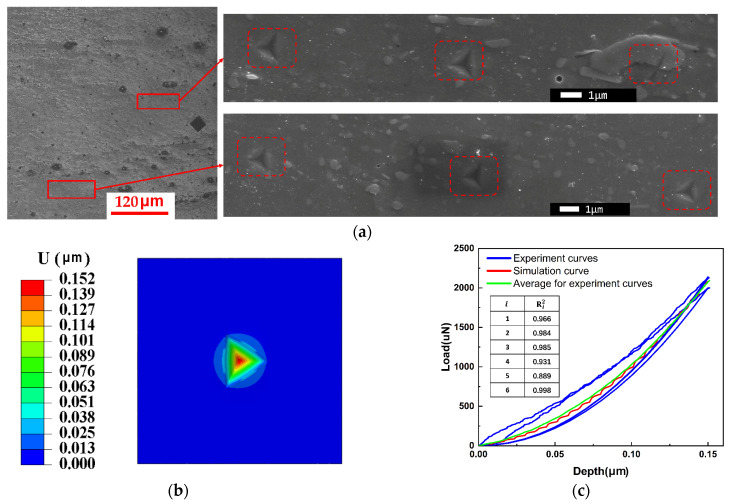
Experimental and simulation results of nanoindentation. (**a**) High magnification of nanoindentation; (**b**) finite element results of nanoindentation; (**c**) load–displacement curves in a nanoindentation test at 150 nm.

In this investigation, the stress–strain curve of ferrite in M50 bearing steel was established through the application of the nanoindentation experimental inversion technique. Figure 13a displays the indentation map generated from the nanoindentation experiment. As can be seen in the figure, due to the random nature of the indentation, not all of them are pressed on ferrite (dark gray area in the figure); they are also on cementite (bright gray area in the figure) or at the junction of the two phases. Moreover, each indentation was separated by a distance of at least 8 µm [33] in order to avoid mutual influence between the indentations. Figure 13b depicts the results of the finite element simulation corresponding to the nanoindentation process. Furthermore, Figure 13c provides a comparison between the load–displacement curves obtained from the simulation and those derived from experimental data. The findings revealed that the simulation curve demonstrated a comparable trend to the experimental curves and corresponded with the experimental load at an indentation depth of 150 nm. Consequently, it can be inferred that the stress–strain curves of ferrite in M50 bearing steel, as determined by the power law model, were fundamentally accurate. In order to ascertain whether the curve predictions met the benchmark requirements, we proposed the concept of goodness of fit, which is now widely used to assess the accuracy of simulation results. The specific method and equation are as follows. The goodness of fit of the simulated or experimental curves to the experimental mean curve was first calculated. Then, the results were compared. The specific results are displayed in the figure. The calculated goodness of fit of the simulated curve to the average curve was the largest (0.998), which exceeded the goodness of fit of the other experimental curves, providing substantial evidence for the accuracy of the curves. However, the plastic and elastic work generated by the indenter cannot be well illustrated by simulation due to the different paths of loading and unloading. Therefore, only the loading process was simulated in this paper. For additional information, please consult Equation (3).

(2) $${R_{i}}^{2}=1-\frac{\sum (y-\hat{y_{i}}{)}^{2}}{\sum (y-\bar{y}{)}^{2}}$$

where $R_{i}^{2}$ is the goodness of fit of the experimental or simulation curve relative to the experimental mean curve, i = 1~6. When i = 1~5, it is five sets of experimental curves. When i = 6, it is the simulation curve. y is the load value of the experimental mean curve, $\hat{y_{i}}$ is the load value of the experimental or simulation curve, and $\bar{y}$ is the mean value of the load of the experimental mean curve.

(3) $$\sigma =\left\{\begin{array}{l} E\cdot \varepsilon ,\sigma <\sigma_{p}\\ R\cdot \varepsilon_{p}^{n},\sigma \geq \sigma_{p} \end{array}\right.$$

where the modulus of elasticity is 205 GPa, the yield strength is 376.5 MPa, the hardening coefficient R is 1155.75, and the hardening index n is 0.178.

**Figure 14:**
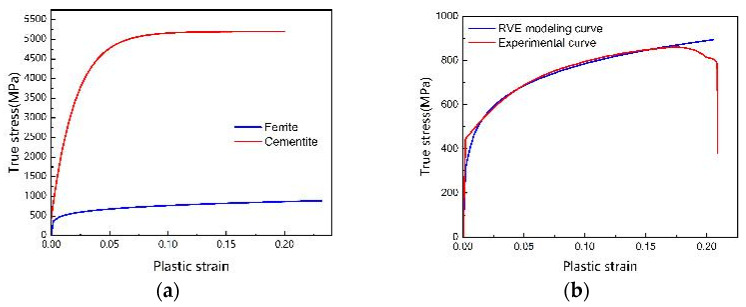
M50 bearing steel parameter curves. (**a**) Single-phase stress–strain curve; (**b**) RVE single-phase tensile validation plot.

As noted by Bhadeshia [34], the modulus of elasticity for cementite was reported to be 230 GPa, with a Poisson’s ratio of 0.3. The stress–strain behavior of cementite can be characterized by a non-linear equivalent stress–strain relationship, which was mathematically represented in an exponential form, as delineated in Equation (4) below.

(4) $$\sigma_{p}=\sigma_{p}^{0}+(\sigma_{p}^{1}-\sigma_{p}^{0})(1-\exp^{-\frac{E_{\theta }}{\sigma_{p}^{1}-\sigma_{p}^{0}}\varepsilon_{nl}})$$

where $\sigma_{p}^{0}$ can be taken as the yield strength of the steel, which is 444.52 MPa, as seen in Figure 14b. According to the simulations of the cementite by Hu et al. [35], $\sigma_{p}^{1}$ is 5.2 GPa, $\varepsilon_{nl}$ is the inelastic strain, and $\sigma_{p}$ is the equivalent stress in the cementite. The ultimate stress–strain curve for ferrite and cementite is depicted in Figure 14a. Figure 14b provides a comparative analysis of the curves obtained from unidirectional stretching conditions applied to the RVE alongside the corresponding experimental curves. As illustrated, there was a notable degree of overlap between the two sets of curves, which served to validate the efficacy of the RVE model utilized in this research.

**Figure 15:**
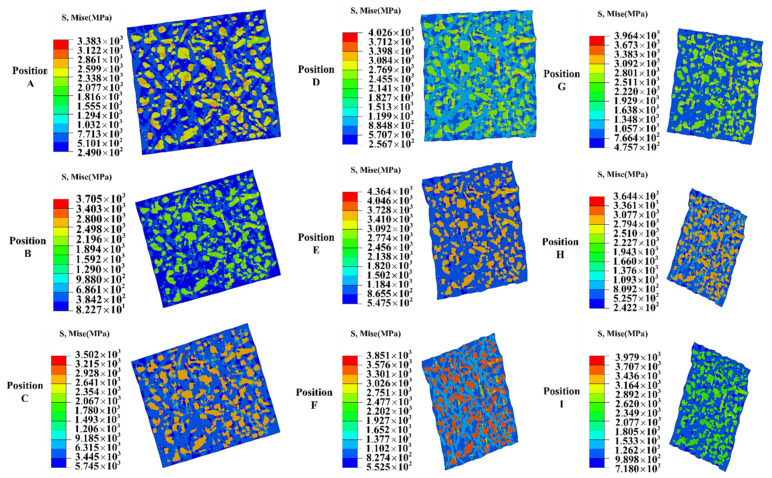
RVE stress distribution at different locations of the M50 bearing ring at the end of rolling.

**Figure 16:**
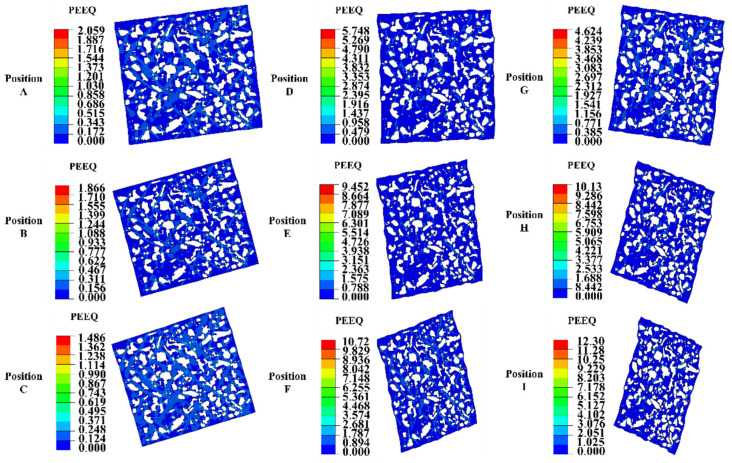
RVE strain distribution at different locations of the M50 bearing ring at the end of rolling.

Figure 15 and Figure 16 illustrate the mesoscale stress–strain distribution corresponding to various positions on the M50 cold-rolled bearing ring, specifically aligned with the nine locations identified in Figure 12. As depicted in Figure 15, significant disparities in stress distribution between the ferrite and cementite phases were noted. The highest concentration of stress was observed at the interface of these two phases, which may facilitate the development of micropores and microcracks within the material matrix during the rolling deformation process [36]. According to Figure 16, deformation first happened in the ferrite close to the cementite–ferrite interface, and its rate increased as the distance from the cementite decreased. The ferrite farther away from this interaction was then affected by the deformation. Most of the plastic strains were found in the ferrite, specifically in the area with the highest strain, which was mostly found in the ferrite matrix between two neighboring cementite particles. This area was likely to encounter significant challenges related to deformation coordination. Additionally, the degree of RVE deformation at each feature location showed variability, as shown in Figure 15 and Figure 16. The RVE’s deformation was greater in the ring’s middle thickness region than it was at the upper-end face. The radial height of both areas is the same. This observation aligns with observations made at the macroscopic level. Additionally, the deformation of the RVE in the inner surface region of the ring at the same axial height was more pronounced than that at the outer surface, with the maximum radial deformation of the RVE consistently occurring at the raceway.

**Figure 17:**
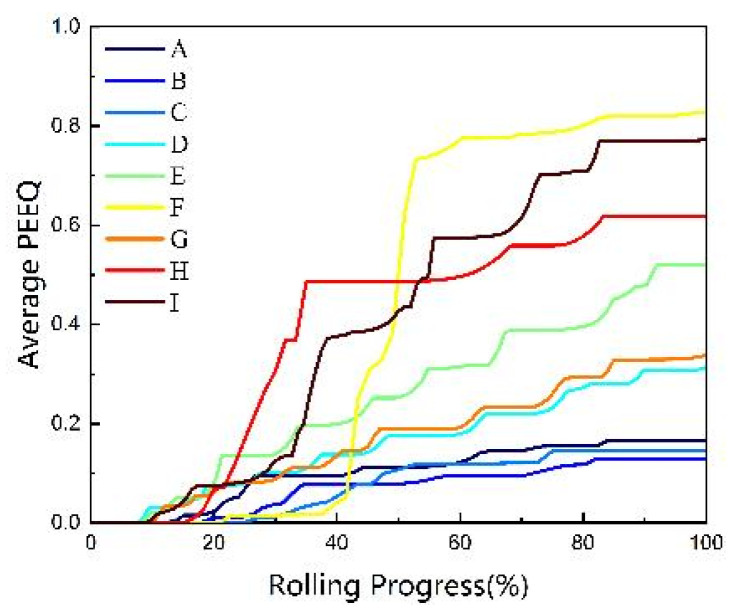
Ferrite mean PEEQ curve.

The average PEEQ variation curves for ferrite in the RVE at various places (A~I) of the M50 bearing ring are displayed in Figure 17. The deformation results at the end of rolling were basically consistent with the macro-PEEQ curves. The PEEQ at each location was practically zero because the ferrite was still in the elastic deformation stage at the beginning of the rolling process. As the rolling process continued, the soft ferrite phase began to gradually experience plastic deformation. However, the average strain of ferrite in the entire RVE varies due to the various strains applied to each RVE. After rolling, the ring’s upper surface position had the highest radial average strain of the outer surface element, while the other locations had the highest average PEEQ of the inner surface element. The medium thickness area element showed the lowest average strain in all positions. The reason why the upper surface position appeared different from the others could be that the material flow caused the area to not fit the mandrel roll entirely, which decreased the amount of rolling force delivered to the area and, as a result, the degree of deformation. Axial patterns were observed on the outer surfaces (A, D, G) and middle thickness (B, E, H), with the PEEQ decreasing as the distance from the ring’s center increased. On the inner surface of the ring element, the strain was highest at F and the lowest at C. The F spot was situated at the raceway’s edge, where the shear force was higher and a greater shear deformation took place. This led to the conclusion that shear was another significant element in the ring’s deformation.

**Figure 18:**
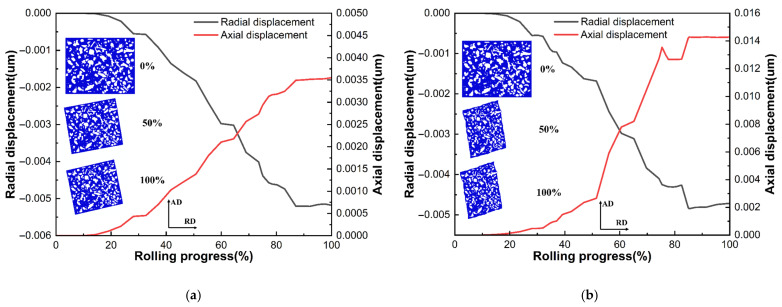

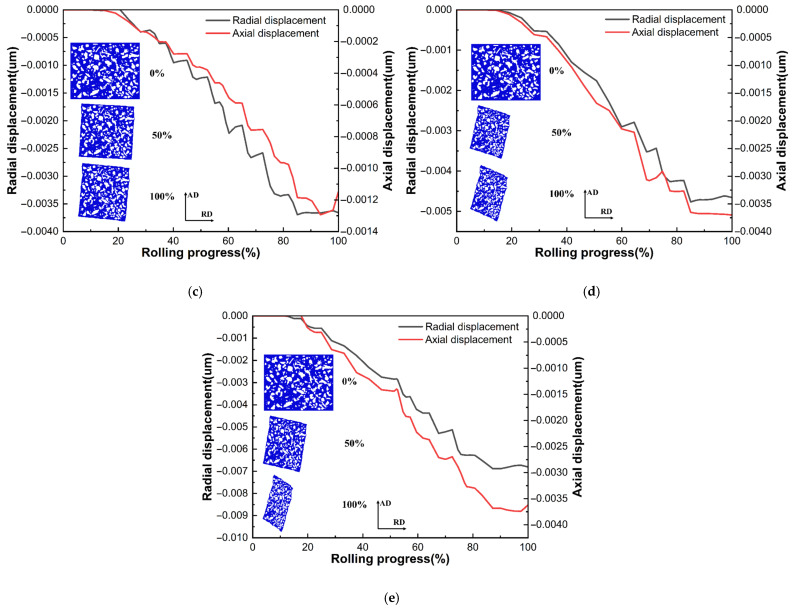
Variation curves of cementite displacement in the radial–axial plane: (**a**) position C; (**b**) position F; (**c**) position G; (**d**) position H; (**e**) position I.

Based on the findings of the aforementioned analyses, the elements in the medium-height region and those on the inner surface of the ring were chosen for investigation. Figure 18 shows the cementite flow curves and deformation of the RVE, where Figure 18a,b,e show the axial elements of the ring and Figure 18c–e show the radial elements. As shown in the figure, the cementite essentially did not experience displacement changes at the beginning of the rolling process because the ferrite was still in the elastic deformation stage. As rolling continued, the ferrite gradually experienced plastic deformation and flowed. For axial and radial movement, the ferrite was wrapped around the cementite. The RVE displayed a compressed state for the axial element, and the cementite flowed radially in the direction of the RVE compression. Displacement was observed to be maximal at the edge of the raceway and minimal at the upper surface of the ring. The shear force exerted a considerable influence on the RVE in the axial direction, leading to a specific angle of deflection and reduced stretching. The cementite at the upper-end face and the edge of the raceway showed the flow trend along the deflection direction, whereas the cementite in the middle of the raceway was subjected to the opposite shear force and showed the opposite flow trend. All three had nearly identical axial displacements. The flow behavior for axial and radial elements was similar in the radial direction. As the RVE was subjected to shear forces in the same direction in the axial direction, the cementite flowed in the shear direction. The inner surface and the middle width region showed notable cementite displacements, whereas the outer surface showed only slight displacements. This phenomenon can be attributed to the outer surface of the ring being subjected to greater radial forces and lesser shear forces, which resulted in minimal changes in the RVE in the axial direction. However, the RVE deformed more severely in the axial direction at other places of the ring due to significant shear stresses.

**Figure 19:**
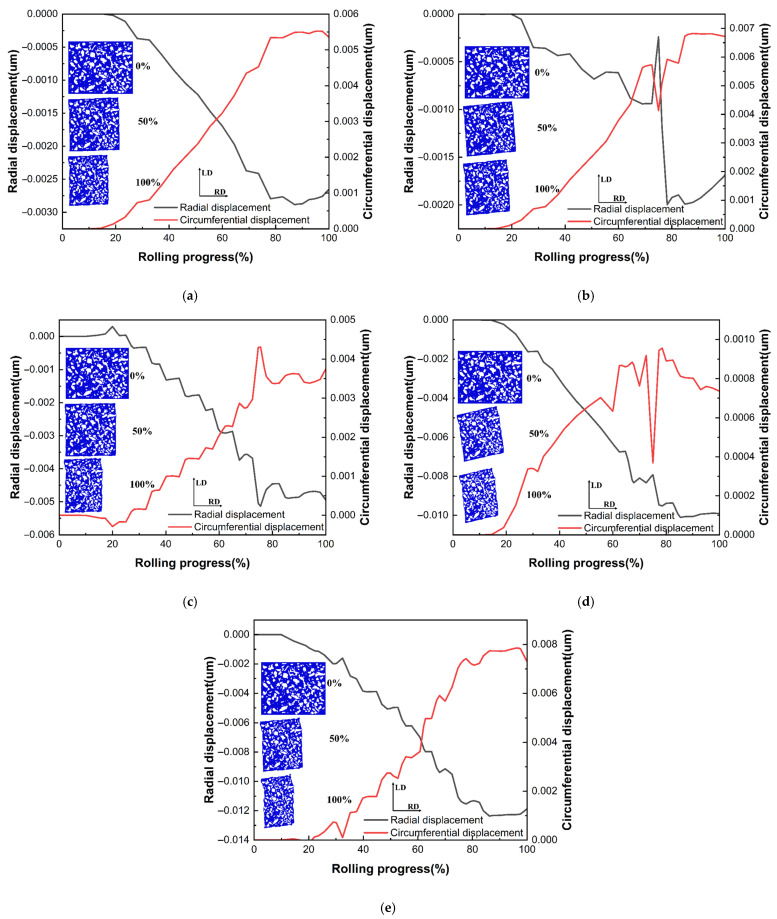
Variation curves of cementite displacement in the radial–circumferential plane: (**a**) position C; (**b**) position F; (**c**) position G; (**d**) position H; (**e**) position I.

Figure 19 shows the distribution and displacement change curves of the RVE in the radial–circumferential direction for each of the above positions. As observed in the image, similar to the radial–axial direction, the flow of cementite basically did not occur in the early stages of ring rolling, and cementite was wrapped by ferrite for flow in the middle and late stages of rolling. The axial elements (Figure 19a,b,e) experienced both circumferential and radial forces. In the radial direction, the displacement of the cementite at the center of the raceway was clearly the largest, but the displacement at other positions was relatively small. The displacement of the cementite in the circumferential direction showed a tendency to increase gradually from the upper-end surface of the ring to the center of the ring raceway. This was because the ring’s circumferential flow increased with proximity to the raceway. For the radial elements (Figure 19c–e), the cementite’s circumferential displacement showed a tendency to gradually decrease from the inner and outer surfaces to the middle thickness region of the ring, while its radial displacement showed a tendency to gradually increase from the outer surface to the inner surface of the ring.

### 3.3. Mechanisms of Macroscale and Mesoscale Flow Behavior Evolution

**Figure 20:**
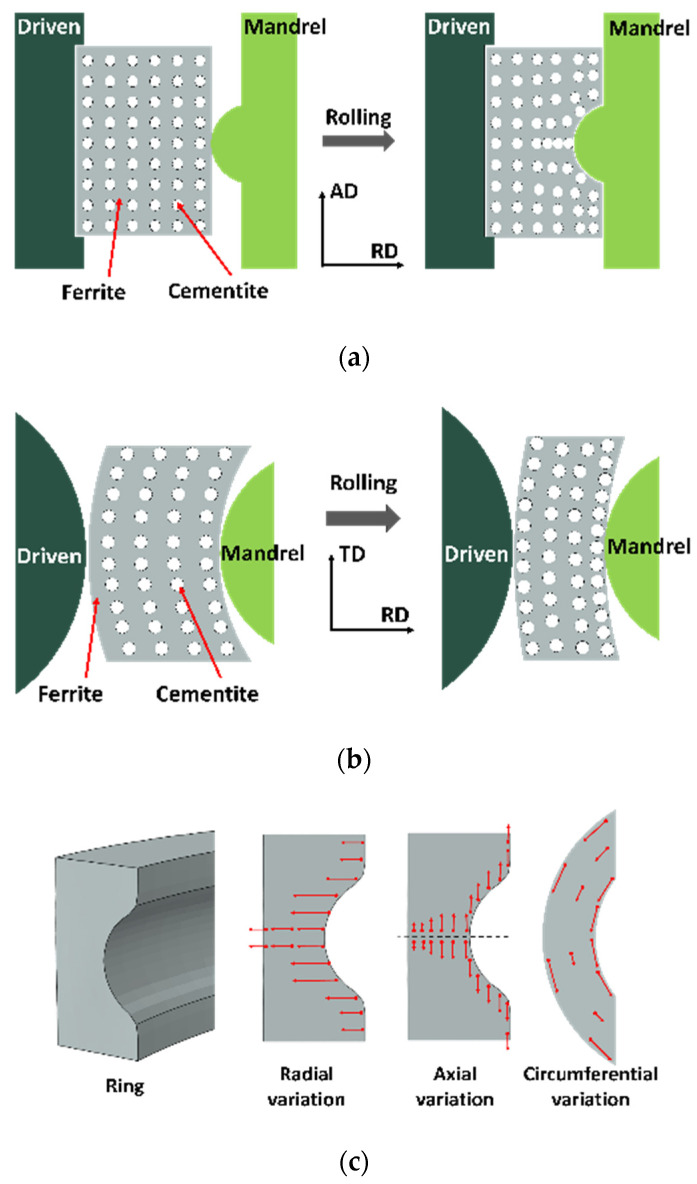
Mechanism of macro- and microflow behavior during the ring rolling process. (**a**) Radial–axial direction; (**b**) radial–circumferential direction; (**c**) schematic of the flow behavior after rolling.

Figure 20 shows the mechanism of macroscopic and mesoscopic flow behavior during cold rolling. It is clear that the cementite dispersion is irregular with the mandrel roll feed during the ring rolling process. As seen in Figure 20a, the cementite displacement on the inner surface of the ring is substantially larger than on the outer surface of the ring in the radial direction. In the axial direction, the cementite at the ring raceway exhibits a large displacement because of the high shear force. Additionally, when the cementite advances away from this region, its axial displacement reduces. As illustrated in Figure 20b, the displacement of cementite on the inner surface of the ring is larger than that on the outer surface in the radial direction in the radial–circumferential plane. In the circumferential direction, the figure shows more cementite at the ring’s inner and outer surfaces than in the middle region due to greater displacement at the surfaces. Some shapes are defined in Figure 20c, where the circle indicates the position of the initial cementite, the red arrow indicates the direction of displacement, and the length of the line segment indicates the relative magnitude of the displacement distance. As shown in Figure 20c, the displacement of the cementite shows a certain pattern. The flow of the cementite is most intense in the radial direction in the raceway edge region, followed by the raceway center region, and finally, other regions. And, at the middle height of the ring, the displacement of the cementite gradually decreases from the inner surface to the outer surface. The displacement of cementite at the ring’s middle height gradually decreases from the inner to the outer surface. However, on the inner surface, the displacement remains essentially unchanged along the axial direction. The circumferential variation of the cementite is shown by the fact that the cementite on the inner and outer surfaces of the ring maintains approximately the same displacement, while the cementite in the middle thickness region of the ring is less displaced.

This paper successfully combines macro- and mesoviews to reflect the flow trend of cementite in the cold ring rolling process. The method is of great significance in guiding the bearing-forming process. Through this method, the macroscopic rolling and forming process can be designed so that different areas of the bearing can be pretreated differently at the early stage of rolling or even at the stage of raw material acquisition. Then, the ideal bearing properties can be obtained in the area through the rolling process so as to facilitate the subsequent heat treatment process. Specifically, the initial material can be optimized according to the stress and strain distribution. Through reasonable heat treatment of the initial material, the stress concentration phenomenon in the cold ring rolling process can be reduced, and the damage in the cold ring rolling process can be reduced or avoided. In addition, this method is not only applicable to the cold ring rolling process of M50 bearing steel but also to the forming process of other high-carbon steels.

## 4. Conclusions

This paper established a multiscale model from the macroscale to the mesoscale. Based on the actual working conditions, a macroscopic cold-rolled three-dimensional finite element model of the M50 bearing ring was established. The sub-model was applied to transfer the response between macroscale and mesoscale. Based on the actual microstructure of the material, the RVE model was developed and verified. Through nanoindentation studies, the material’s single-phase characteristics were determined. A mesoscale finite element model was finally developed. Analysis was performed on the cold ring rolling macroscale and mesoscale flow characteristics of M50 bearing steel. This approach significantly lowers the time and computational resources needed for direct full-scale modeling while preserving the capture of the high-precision results in crucial regions using the sub-modeling technique to decouple the macroscopic and mesoscale computations. The conclusions of this paper are as follows:

1. Through the macroscopic cold ring rolling three-dimensional finite element simulation, it was found that the stress distribution was relatively uniform. The PEEQ showed a stepwise growth. In the axial direction, the PEEQ showed a trend of high in the middle and low on both sides, and in the radial direction, it showed a trend of high in the middle of the two ends. The maximum PEEQ appeared in the raceway.
2. The mesoscopic simulation revealed that the stress was concentrated in the cementite, and the maximum stress occurred at the junction of the two phases. The greatest strain was found in the ferrite matrix positioned between the two adjacent cementites, while the strain first appeared in the matrix at the cementite–ferrite junction.
3. The cementite flew with the deformation of the matrix. The radial displacement of the cementite decreased from the edge of the raceway to both ends and decreased from the inner to the outer surface. Its axial displacement was basically the same on the inner surface of the ring and decreased from the inner to the outer surface. Its circumferential displacement decreased from the inner and outer surfaces to the intermediate thickness region.

## Figures and Tables

**Table 1:** Chemical composition of M50 bearing steel (wt.%).

C	Cr	Mo	V	Ni	Mn	Si	Fe
0.8	4.02	4.2	0.93	0.05	0.29	0.16	Bal.

**Table 2:** M50 bearing ring cold rolling parameters.

Parameter	Value
Blank size/(mm × mm × mm)	Φ41 × 29 × 10
Ring size/(mm × mm × mm)	Φ55 × 45 × 10
Depth of groove ball pressing in/mm	2
Driven roller radius/mm	25
Rotate speed of driven roller/rad∙s^−1^	6.3
Mandrel roller radius/mm	10
Feeding speed of mandrel roller/mm∙s^−1^	2
Guide roller radius/mm	10

**Table 3:** Finite element simulation dimensions of the forging at the end of rolling and expected dimensions.

Parameters	Simulation Size (mm)	Expected Size (mm)	Difference (%)
OD	54.794~54.915	55	−0.375~−0.015
ID	44.856~44.949	45	−0.032~−0.013
Thickness	4.975~4.996	5	−0.5~−0.008

## Data Availability

The original contributions presented in this study are included in the article. Further inquiries can be directed to the corresponding authors.

## References

[1] Rejith R., Kesavan D., Chakravarthy P., Murty S.N. (2023). Bearings for aerospace applications. Tribol. Int..

[2] Bhadeshia H.K.D.H. (2012). Steels for bearings. Prog. Mater. Sci..

[3] Zaretsky E.V. (2012). Rolling bearing steels—A technical and historical perspective. Mater. Sci. Technol..

[4] Sun B., Xu J., Xing C. (2019). Numerical and experimental investigations on the effect of mandrel feeding speed for high-speed rail bearing inner ring. Int. J. Adv. Manuf. Technol..

[5] Qian D., He Y., Wang F., Chen Y., Lu X. (2020). Microstructure and mechanical properties of M50 steel by combining cold rolling with austempering. Metals.

[6] Wang F., Qian D., Xie L., Dong Z., Song X. (2021). Microstructure evolution and tempering transformation kinetics in a secondary hardened M50 steel subjected to cold ring rolling. ISIJ Int..

[7] Wang F., Qian D., Hua L., Lu X. (2019). The effect of prior cold rolling on the carbide dissolution, precipitation and dry wear behaviors of M50 bearing steel. Tribol. Int..

[8] Deng S., Hua L., Shi D. (2017). Effect of cold rolling on plastic deformation and microstructure of bearing ring. Mater. Sci. Technol..

[9] Li W., Sakai T., Li Q., Lu L.T., Wang P. (2010). Reliability evaluation on very high cycle fatigue property of GCr15 bearing steel. Int. J. Fatigue.

[10] Wu H.N., Xu D.S., Wang H., Yang R. (2016). Molecular dynamics simulation of tensile deformation and fracture of γ-TiAl with and without surface defects. J. Mater. Sci. Technol..

[11] Gadalińska E., Baczmański A., Braham C., Gonzalez G., Sidhom H., Wroński S., Buslaps T., Wierzbanowski K. (2020). Stress localisation in lamellar cementite and ferrite during elastoplastic deformation of pearlitic steel studied using diffraction and modelling. Int. J. Plast..

[12] Lu X., Zhou L., Lei C., Liu H., Lan H. (2023). Formation mechanism and microstructure evolution of light etched region during rolling contact fatigue in M50 steel. J. Mater. Res. Technol..

[13] Zhang C., Peng B., Wang L., Ma X., Gu L. (2019). Thermal-induced surface damage of M50 steel at rolling-sliding contacts. Wear.

[14] Guan J., Wang L., Zhang Z., Shi X., Ma X. (2018). Fatigue crack nucleation and propagation at clustered metallic carbides in M50 bearing steel. Tribol. Int..

[15] Ganti S., Turner B., Kirsch M., Anthony D., McCoy B., Trivedi H., Sundar V. (2018). Three-dimensional (3D) analysis of white etching bands (WEBs) in AISI M50 bearing steel using automated serial sectioning. Mater. Charact..

[16] Macioł P., Michalik K. (2016). Parallelization of fine-scale computation in Agile Multiscale Modelling Methodology. AIP Conf. Proc..

[17] Su Y., Shen Z., Long X., Chen C., Qi L., Chao X. (2023). Gaussian filtering method of evaluating the elastic/elasto-plastic properties of sintered nanocomposites with quasi-continuous volume distribution. Mater. Sci. Eng. A.

[18] Kong L., Xie H., Li C. (2023). Coupled microplane and micromechanics model for describing the damage and plasticity evolution of quasi-brittle material. Int. J. Plast..

[19] Zhang J., Li H., Sun X., Zhan M. (2020). A multi-scale MCCPFEM framework: Modeling of thermal interface grooving and deformation anisotropy of titanium alloy with lamellar colony. Int. J. Plast..

[20] Zhou J., Gokhale A.M., Gurumurthy A., Bhat S.P. (2015). Realistic microstructural RVE-based simulations of stress–strain behavior of a dual-phase steel having high martensite volume fraction. Mater. Sci. Eng. A.

[21] Ji H., Song Q., Gupta M.K., Liu Z. (2020). A pseudorandom based crystal plasticity finite element method for grain scale polycrystalline material modeling. Mech. Mater..

[22] Chawla N., Sidhu R.S., Ganesh V.V. (2006). Three-dimensional visualization and microstructure-based modeling of deformation in particle-reinforced composites. Acta Mater..

[23] Jang J.I., Shim S.H., Komazaki S., Sugimoto T. (2006). Correlation between microstructure and nanohardness in advanced heat-resistant steel. Key Eng. Mater..

[24] Yan F.K., Zhang B.B., Wang H.T., Tao N.R., Lu K. (2016). Nanoindentation characterization of nano-twinned grains in an austenitic stainless steel. Scr. Mater..

[25] Hu X., Yang L., Wei X., Wang H., Fu G. (2023). Molecular dynamics simulation on nanoindentation of M50 bearing steel. Materials.

[26] Cheng G., Choi K.S., Hu X., Sun X. (2016). Determining individual phase properties in a multi-phase Q&P steel using multi-scale indentation tests. Mater. Sci. Eng. A.

[27] Young M.L., Almer J.D., Daymond M.R., Haeffner D.R., Dunand D.C. (2007). Load partitioning between ferrite and cementite during elasto-plastic deformation of an ultrahigh-carbon steel. Acta Mater..

[28] Taupin V., Pesci R., Berbenni S., Berveiller S., Ouahab R., Bouaziz O. (2013). Lattice strain measurements using synchrotron diffraction to calibrate a micromechanical modeling in a ferrite–cementite steel. Mater. Sci. Eng. A.

[29] Oliver W.C., Pharr G.M. (1992). An improved technique for determining hardness and elastic modulus using load and displacement sensing indentation experiments. J. Mater. Res..

[30] Zhuang X., Ma S., Zhao Z. (2016). Effect of particle size, fraction and carbide banding on deformation and damage behavior of ferrite–cementite steel under tensile/shear loads. Model. Simul. Mater. Sci. Eng..

[31] Ma S., Zhuang X., Zhao Z. (2016). Effect of particle size and carbide band on the flow behavior of ferrite–cementite steel. Steel Res. Int..

[32] Zhao K., Zhuang X.C., Pei X.H., Zhao Z. (2017). Influence of Geometrical Imperfection on Failure Mode of DP780 Steel Utilizing Damage Models Embedded RVE Technique. Key Eng. Mater..

[33] Viloria A., Nova D.M., Salinas D.A.G., Barbosa W., Espinosa C.C.P., Toledo F.A.R., Ballesteros D.Y.P., Rodriguez J.G.D. (2024). Microhardness Profile and Residual Stresses Evaluation in a Shot Peened SAE 5160H Steel. Rev. UIS Ing..

[34] Bhadeshia H. (2020). Cementite. Int. Mater. Rev..

[35] Hu X., Van Houtte P., Liebeherr M., Walentek A., Seefeldt M., Vandekinderen H. (2006). Modeling work hardening of pearlitic steels by phenomenological and Taylor-type micromechanical models. Acta Mater..

[36] Wang H., Wang F., Qian D., Chen F., Dong Z., Hua L. (2023). Investigation of damage mechanisms related to microstructural features of ferrite-cementite steels via experiments and multiscale simulations. Int. J. Plast..

